# MAFLD progression contributes to altered thalamus metabolism and brain structure

**DOI:** 10.1038/s41598-022-05228-5

**Published:** 2022-01-24

**Authors:** Saverio Nucera, Stefano Ruga, Antonio Cardamone, Anna Rita Coppoletta, Lorenza Guarnieri, Maria Caterina Zito, Francesca Bosco, Roberta Macrì, Federica Scarano, Miriam Scicchitano, Jessica Maiuolo, Cristina Carresi, Rocco Mollace, Luca Cariati, Giuseppe Mazzarella, Ernesto Palma, Micaela Gliozzi, Vincenzo Musolino, Giuseppe Lucio Cascini, Vincenzo Mollace

**Affiliations:** 1 Institute of Research for Food Safety and Health IRC-FSH, Department of Health Sciences, University Magna Graecia of Catanzaro, 88100 Catanzaro, Italy; 2 Nuclear Medicine Unit, Department of Diagnostic Imaging, University Magna Graecia of Catanzaro, 88100 Catanzaro, Italy

**Keywords:** Biochemistry, Dyslipidaemias, Metabolic syndrome, Non-alcoholic fatty liver disease, Non-alcoholic steatohepatitis, Magnetic resonance imaging

## Abstract

Metabolic associated fatty liver disease (MAFLD), commonly known as non-alcoholic fatty liver disease, represents a continuum of events characterized by excessive hepatic fat accumulation which can progress to nonalcoholic steatohepatitis (NASH), fibrosis, cirrhosis, and in some severe cases hepatocellular carcinoma. MAFLD might be considered as a multisystem disease that affects not only the liver but involves wider implications, relating to several organs and systems, the brain included. The present study aims to investigate changes associated with MAFLD-induced alteration of thalamic metabolism in vivo. DIAMOND (Diet-induced animal model of non-alcoholic fatty liver disease) mice were fed a chow diet and tap water (NC NW) or fat Western Diet (WD SW) for up to 28 weeks. At the baseline and weeks 4, 8, 20, 28 the thalamic neurochemical profile and total cerebral brain volume were evaluated longitudinally in both diet groups using ^1^H-MRS. To confirm the disease progression, at each time point, a subgroup of animals was sacrificed, the livers excised and placed in formalin. Liver histology was assessed and reviewed by an expert liver pathologist. MAFLD development significantly increases the thalamic levels of total N-acetylaspartate, total creatine, total choline, and taurine. Furthermore, in the WD SW group a reduction in total cerebral brain volume has been observed (*p* < 0.05 vs NC NW). Our results suggest that thalamic energy metabolism is affected by MAFLD progression. This metabolic imbalance, that is quantifiable by ^1^H-MRS in vivo*,* might cause structural damage to brain cells and dysfunctions of neurotransmitter release.

## Introduction

Metabolic (dysfunction)-associated fatty liver disease (MAFLD)^1,2^ formerly known as non-alcoholic fatty liver disease (NAFLD)^3^ is a heterogeneous condition of fatty liver disease which might be influenced by multiple factors including age, gender, hormonal status, ethnicity, diet, alcohol intake, smoking, genetic predisposition, the microbiota and metabolic status^4^. The spectrum of the disease extends from steatosis to hepatocellular carcinoma (HCC)^5^ and though hepatic steatosis is highly prevalent, inflammation occurs only in a minority. Moreover, liver-related complications (i.e., cirrhosis or cancer) are likely in patients with steatohepatitis^6^, but the progression is not inevitable or consequential. Indeed, cirrhosis is not a fundamental stage for HCC development^7^.

This heterogeneity also underlines the possible impact of MAFLD on several organs and systems, included the brain. In fact, nervous dysfunctions^8^, brain lesions, changes in perfusion and brain activity^9^, brain aging, increased risk of ischemic and hemorrhagic stroke^10,11^ are some of the consequences of the wide spectrum of extrahepatic alterations induced by MAFLD.

In particular, oxidative stress, in the disease progression, leads to alterations in mitochondrial function and structure with a consequent reduction in neuronal metabolism^12–14^. In turn, the alteration of metabolic activity in specific brain areas (i.e., thalamus, hippocampus, pre-frontal cortex) could cause cognitive deficits^15^ because various metabolites, such as N-acetylaspartate, creatine, choline, glutamate and taurine^16–19^ are involved in energy metabolism and in the maintenance of brain functions^20^.

Changes in the cerebral levels of those metabolites, following a fatty diet, have been reported in preclinical studies^21^ and evidence exists that the consumption of a high caloric diet also leads to high concentrations of inflammatory cytokines in the brain, resulting in microgliosis, astrocytosis and neuronal damage^8,22^. Moreover, patients suffering from steatohepatitis have a reduced brain volume^23^ and are at higher risk to suffer from neurological diseases, which are, most probably, related to the volume reductions as well^24^.

Altogether, these discoveries provide a rationale to further evaluate the role of MAFLD in brain damage, through the identification, visualization and quantification of brain biochemical markers and neurotransmitters, and the alteration of the brain volume that, overall, could reflect physiologic or pathologic conditions^25^. In this perspective, advances in neuroimaging provide unique opportunities to evaluate brain structure, biochemistry and function^26^. In particular, the proton magnetic resonance spectroscopy (^1^H-MRS) represents a non-invasive method useful to study brain metabolism, longitudinally and in a non-invasive manner^27^.

Therefore, the aim of our work was to analyze the total brain volume in a diet-induced animal model of non-alcoholic fatty liver disease (DIAMOND)^28^ and to assess and quantify the main metabolites present in brain tissue, including total N-acetylaspartate (NAA + NAAG), the predominant MRS signal in the healthy neurons^16^; total creatine (tCr), involved in cellular energy metabolism; total choline (tCho), also involved in the synthesis and the breakdown of cellular membranes^29^; the neuroinflammation modulator taurine (Tau)^30,31^ and the excitatory neurotransmitter glutamate (Glu)^32^. As the concentration of brain metabolites often reflects the state of its metabolic activity and energetic status, the ^1^H-MRS analysis of the metabolic fluctuation might be predictive of the potential MAFLD implications at brain level.

## Materials and methods

### Animals

Male DIAMOND (diet-induced animal model of non-alcoholic fatty liver disease) mice were purchased from Sanyal Biotechnology (Virginia Beach, VA, USA) kept under standard laboratory conditions in a specific‐pathogen-free animal facility and maintained at 22 ± 2 °C with alternating 12 h light–dark cycle. The model is a stable isogenic cross between C57BL/6J (B6) and 129S1/SvImJ (S129) mice. Those mice are prone to recapitulate the key physiological, metabolic, histologic, transcriptomic, and cell-signaling changes seen in humans with progressive NASH only if they are fed with a high fat diet, high carbohydrate diet (Western diet, WD) with 42% kcal from fat and containing 0.1% cholesterol with a high fructose-glucose solution (SW, 23.1 g/L d-fructose + 18.9 g/L d-glucose). Conversely, DIAMOND mice fed with a standard chow diet (NC) with normal water (NW) did not develop the disease^6,28^. All the experimental procedures were performed according to protocols approved by the Animal Care of University Magna Graecia of Catanzaro. The experimental procedures were carried out in compliance with the ARRIVE guidelines. All experiments were performed in accordance with the European Commission guidelines (Directive 2010/63/EU) for the animals used for scientific purposes.

### Study design

Mice of 8–12 weeks of age and weight of 20.54 ± 0.53 g were divided into two groups and fed ad libitum a normal chow diet (NC, Harlan TD.2019) and tap water (NW) or a high fat/high carbohydrate diet (Western diet, WD, Harlan TD.88137) with a high fructose-glucose water solution (SW, 23.1 g/L d-fructose + 18.9 g/L d-glucose) for up to 28 weeks. The choice of the animals and the diet used to develop steatosis and steatohepatitis have been based on previously published studies^6,28^. One day before starting diet regimen, baseline body weight and MRS were assessed. On weeks 4, 8, 20 and 28, thalamic neurochemical profile was evaluated in the two diet cohorts using ^1^H-MRS. Animals body weight was assessed weekly. The day of the sacrifice, animals were exposed to inhaled isoflurane prior to being euthanized. Euthanasia was performed by cervical dislocation. The entire liver was removed from the abdominal cavity and weighed. The liver was sectioned in a sagittal plane and placed in containers of 10% formalin, for later histologic processing and analysis.

### ^1^H-MRS

Before MRS acquisition, mice were anesthetized with 4% of isoflurane (Forane, Abbott) vaporized in O_2_ (flow: 2 l/min). During acquisition, anesthesia was kept between 1, 5 and 2% to maintain the breathing rhythm (SA Instruments, Inc., Stony Brook, NY, USA) between 50 and 80 breaths per minute. Body temperature was monitored and maintained at 37 °C (SA Instruments, Inc., Stony Brook, NY, USA).

^1^H-MRS spectra were acquired with a Bruker Pharmascan 70/16 US 7 T bore MR scanner (Bruker Biospin MRI GmbH, Ettlingen, Germany), equipped with a Bruker's MRI CryoProbe™ with MRI cryocooler, to increase signal-noise-ratio and reach a higher sensitivity than standard room temperature RF coils.

^1^H-MRS acquisition begins with a single-slice, three-axis localizer scan for the purpose of center mouse brain in the imaging field of view. Subsequently, voxel was precisely placed in the thalamus (2 × 2 × 2 mm^3^) according to anatomical landmarks in Axial and Sagittal T2_turboRARE weighted images with fat suppression (TE: 35 ms, TR: 2500 ms, averages: 2, thickness of 0.7 mm, slices 9, field of view 20 × 20 mm^2^, data matrix 256 × 256). The thalamic voxel (2 × 2 × 2 mm^3^) was positioned based upon referencing a mouse brain atlas. A localized shimming procedure was performed to improve the B_0_ field map homogeneity in the region of interest. Then, a variable power and an optimized relaxation delays (VAPOR pulses) were applied as water suppression scheme. ^1^H-MRS suppressed and unsuppressed spectra were acquired with a PRESS_1H sequence with the following parameters: TE 16.6 ms, TE1 8.99 ms, TE2 7.61, TRd 2500 ms, averages 256, dummy scans 2, VOI 2 × 2 × 2 mm^3^.

^1^H-MRS data analysis was performed using TARQUIN 4.3.10, a new accurate and robust modeling algorithm^33^ which allows to quantify the concentrations of metabolites within the voxel. For the absolute metabolite quantifications were used the two Data File provided by the acquisition, the “fid” data file which are referred to the water suppressed spectra and the “fid.refscan” data file which are referred to the water unsuppressed spectra, both coming from the acquisition in the same voxel location.

### Volumetric analysis

Volumetric analysis was performed using OsiriX imaging software (v. 12.0.2, Pixmeo SARL, Switzerland). Total cerebral brain volumes (TCBVs) were estimated using T1_FLASH_3D images in coronal view with a thickness of 112.78 μm per slice, obtained with a T1_FLASH_3D_iso sequence (TE: 8 ms, TR: 50 ms, averages: 1, dummy scans: 20, image size: 133 × 133 × 80, field of view: 15 × 15 × 10 mm^3^). By the "Draw tool" the ROIs were traced on the MRI sections. To improve the viewing of cerebral margins, a Default WL/WW, "French Clut" and a linear opacity table were used. Total brain's area was defined every 3 slices, starting from the olfactory bulbs up to the last part of the cerebellum. Subsequently, "Generate Missing ROIs" function was used to outline total brain area for all slices. Then, "Compute ROI Volume" feature was used to merge the ROIs of the entire brain and estimate its volume.

### Histological analysis

Liver histology was assessed from paraffin-embedded tissue sections stained with hematoxylin and eosin. Histology was reviewed using the NASH-Clinical Research Network (CRN) criteria and fatty liver inhibition of progression (FLIP) algorithm by a liver pathologist.

For each liver slide, the main histological lesions were assessed using the Steatosis-Activity-Fibrosis (SAF) score system^6,34,35^. Steatosis was graded on a scale of 0 to 3 (0: < 5%; 1: 5–33%; 2: 34–66%; 3: > 67%). Grade of activity (0–2) is given by the sum between the presence of ballooning and inflammation. Ballooning hepatocytes was graded as 0 (none), 1 (when few hepatocytes presented a rounded shaped, reticulated, and pale cytoplasm, but with normal dimensions), and 2 (when there is a cluster of prominent ballooning hepatocytes).

The presence of inflammatory foci within the lobule or within the sinusoids, was graded as 0 (none), 1 (< 2 foci per 20 × field), 2 (2–4 foci per 20 × field), and 3 (> 4 foci per 20 × field). The NAFLD activity score (NAS) was calculated by addition of grades of steatosis, inflammation, and ballooning^5^. Steatohepatitis has been diagnosed as previously described^28^.

### Immunofluorescence

Formalin-fixed, paraffin-embedded sections (5 µm thick) were deparaffinized by exposure to xylene and graded alcohols and then washed in water. Epitopes were retrieved by heating the slides to 98 °C for 30 min in 10 mM Sodium Citrate buffer with pH = 6.0. Sections were washed 3 times with phosphate-buffered saline (PBS) 1X and then permeabilized with a PBS 1X/gelatin (0.2% *w*/*v*)/Triton (0.25% *v*/*v*) solution, twice for 10 min. Subsequently, slides were blocked in a 5% of bovine serum albumin (BSA) (A7906, Sigma Aldrich, Milan, Italy) blocking solution for 1 h at RT and then incubated overnight at 4 °C in a humidified chamber with the 1% BSA-diluted primary antibodies: anti-Ionized calcium-binding adapter molecule 1 (IBA1) antibody, a microglial and macrophage-specific calcium-binding protein involved in the membrane ruffling and phagocytosis in activated microglia, ab178846, Abcam, Cambridge, UK, 1:300; anti- Transmembrane Protein 119 (TMEM119) antibody, a cell-surface protein and a specific microglial marker which has the advantage of distinguishing microglia from macrophages, ab209064, Abcam, Cambridge, UK, 1:200; anti-Glial fibrillary acidic protein (GFAP) antibody, a marker for the activation of astrocytes in the central nervous system, G3893, Sigma Aldrich, Milan, Italy, 1:200; anti-β III Tubulin (TUBB3) antibody which is widely used as a neuron-specific marker, MAB1637, Sigma Aldrich, Milan, Italy, 1:250. After the incubation period, each slide was twice washed with PBS 1X and, after a permeabilization step, incubated with a fluorescent secondary antibody (Alexa Fluor 488 (green), A11029; Alexa Fluor 594 (red), A21203, Thermo Fisher Scientific, Italy) for 1 h at RT. After the washing step, nuclei were stained with DAPI (0.1 µg/ml, D8417, Sigma Aldrich, Milan, Italy) for 10 min at RT.

### Immunofluorescence-derived image acquisition and skeleton analysis

Fluorescence was detected using a confocal laser scanning microscope TCS SP5 (Leica Microsystems, Wetzlar, Germany). Confocal images were acquired using a 63× objective. 180× magnification was used to obtain better details for all processes. For skeleton analysis, IBA1 positive and TMEM119 positive images were used to visualize all microglia processes, GFAP positive images were used to visualize all astrocyte processes, whereas TUBB3 positive images were used to selectively mark the neurons.

Using ImageJ (version 2.1.0/1.53c), the minimum threshold value (IBA1: 90; TMEM119: 100; GFAP: 110; TUBB3: 110) was picked and kept constant between matching NC NW and WD SW thalamic regions. A noise de-speckling was performed to eliminate single-pixel background fluorescence. Then, the resulting images were converted in binary using “Skeletonize” tool. The analysis technique is based on ImageJ plugin AnalyzeSkeleton (2D/3D) (https://imagej.net/plugins/analyze-skeleton/), that provide an analysis of microglia and astrocytes ramification within entire photomicographs. This plugin was applied on the skeletonized images for collect the data regarding total number of process endpoints and summed process length both for microglia and astrocytes. These data were used as measures of microglia, astrocytes, and neurons morphology. In addition, the changes in neuronal network were analyzed using ImageJ performing a measurement of total area of neurons (Soma + neurite) in TUBB3 positive images.

### Statistical analysis

Data were analyzed with GraphPad PRISM 9.1.2 (GraphPad Software, Inc., La Jolla, CA, USA). All results were expressed as mean ± S.D. (Standard deviation). Normality was tested using Shapiro–Wilk normality test. Normally distributed data were analyzed by one way ANOVA followed by Tukey's test, while data without normal distribution were analyzed using Kruskal–Wallis analysis of variance and subsequent Dunn's tests. The Unpaired Two-tailed Student’s *t* test was used for comparison of data derived from two groups. Value with *p* < 0.05 were considered statistically significant. Correlation analysis was assessed using Spearman’s correlation coefficient, using NAS as pathology marker.

## Results

### Mice fed a high fat diet and sugar water develop MAFLD

**Figure 1:**
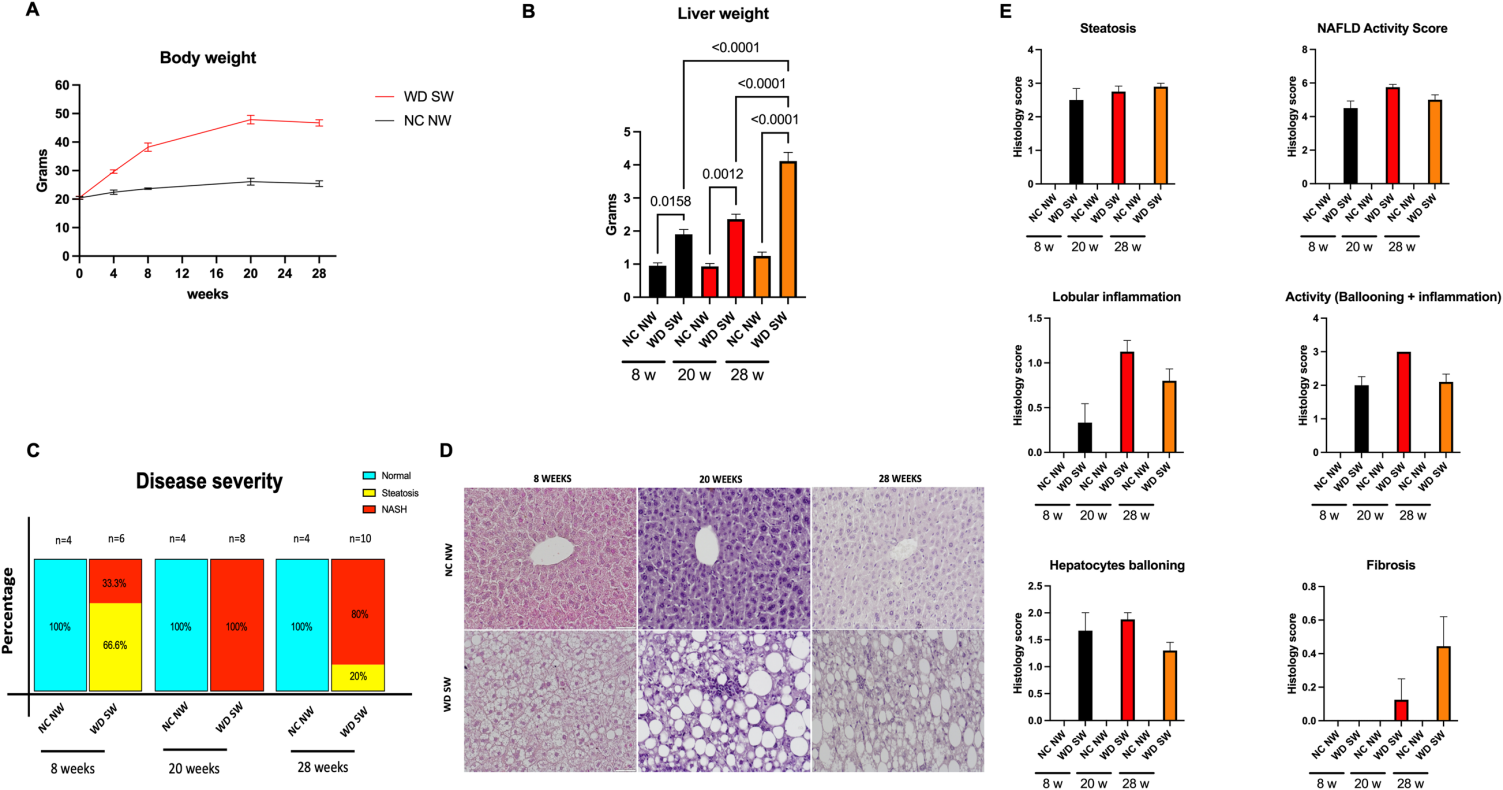
High-fat diet-fed mice develop fatty liver and steatohepatitis. **(A**) body changes over time. Animals were fed a normal chow diet and tap water (NC NW) or high fructose/glucose, high fat Western Diet (WD SW) for up to 28 weeks. (**B**) High fat Western Diet was associated with a significant increase in terminal liver weight compared to CD NW-fed animals at 8, 20 and 28 weeks. (**C**) Disease severity expressed in percentage. (**D**) Gross liver from DIAMOND mice fed a control diet (NC NW) or high fat Western Diet (WD SW) for 8, 20 and 28 weeks. Representative liver sections stained with hematoxylin–eosin (H&E) of livers from CD NW or WD SW mice at 8, 20 or 28 weeks of diet are shown. Original magnification, × 20. (**E**) Histology score for steatosis, hepatocyte ballooning, lobular inflammation, fibrosis and NAFLD Activity Score were quantified. Data are expressed as the mean ± SEM for 4–10 mice per group. For body weight NC NW AUC = 683.1 ± 20.2; WD SW AUC = 1131 ± 50.2. WD SW AUC < 0.001 vs NC NW AUC.

Animals fed a WD SW developed obesity compared to CD NW-fed mice (Fig. 1A). The weight gain was accompanied by an increase in liver weight at all time points (Fig. 1B).

Mice on a high fat diet with ad libitum sugar water administration developed steatohepatitis (Fig. 1C, D), which was characterized by steatosis, lobular inflammation, and hepatocellular ballooning (Fig. 1D, E). The NAFLD activity score (NAS) increased by week 8 and remained higher than NC NW mice by week 28 (Fig. 1E). Specifically, histology of the liver showed an extensive development of steatosis by week 8 in WD SW-fed mice (Fig. 1D, E). At 8 weeks mice had a mean steatosis grade of 2.5 ± 0.3 (Fig. 1E) with 60 ± 11% cells with steatosis (not shown). This remained nearly constant after 20 weeks, with a mean steatosis grade of 2.7 ± 0.1 (Fig. 1E), and 68.75 ± 4.7% cells with steatosis (not shown). At week 28 weeks mice had a mean steatosis grade of 2.9 ± 0.1 (Fig. 1E) and 81 ± 3.4% cells with steatosis (not shown). Following initiation of WD SW diet, steatohepatitis developed in 2 out of 6 mice (33.3%) and at week 20, 8 out of 8 mice (100%) had NASH with a prominent inflammation (Fig. 1C–E), whereas at week 28, 8 out of 10 mice (80%) developed steatohepatitis (Fig. 1C). Stage 1 fibrosis was present by week 20 after initiation of the WD SW diet (Fig. 1E). In contrast, none of the animals on chow diet developed MAFLD (Fig. 1C).

### Volumetric analysis of control and high-fat diet-fed animals

Volumetric analysis showed the same total cerebral brain volumes (TCBVs) in both animal cohorts at 0 weeks.

**Figure 2:**
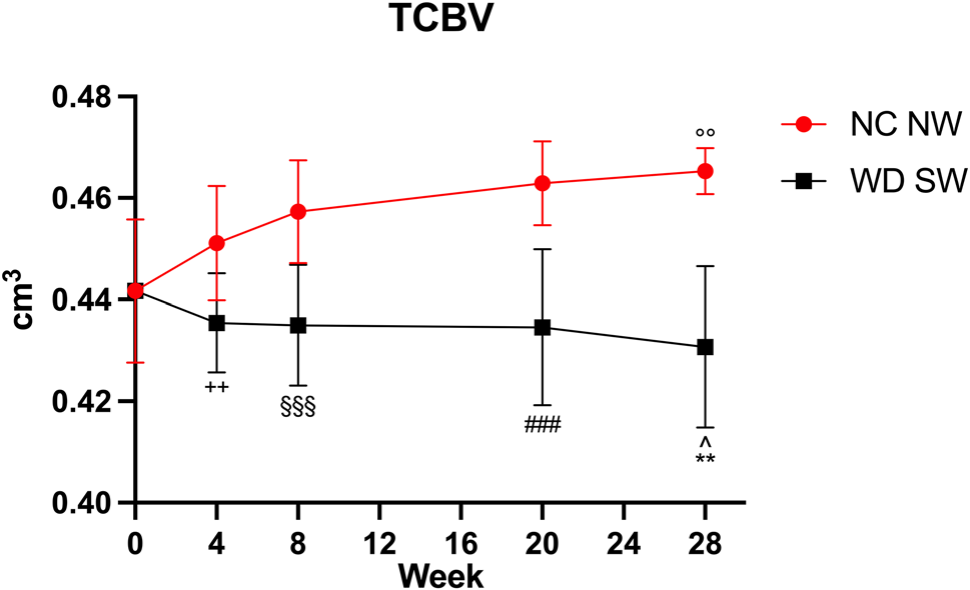
Total cerebral brain volume for the NC NW (red) and WD SW (black) mice during the 24 weeks investigated. T0: 0 weeks, T1: 4 weeks, T2: 8 weeks, T3: 20 weeks, T4: 28 weeks. The results are expressed as mean ± S.D. ^: *p* < 0.05 vs WD SW T0, **: *p* < 0.01 vs NC NW T4, °°: *p* < 0.01 vs NC NW T0, +  + : *p* < 0.01 vs NC NW T1, §§§: *p* < 0.001 vs NC NW T2, ###: *p* < 0.001 vs NC NW T3. NC NW: Normal Chow Normal Water, WD SW: Western Diet Sugar Water.

The results of volumetric analysis showed a statistically significant increase in TCBVs in NC NW group after 28 weeks (0.465 ± 0.004549 cm^3^, *p* < 0.01, +5.34%) (Fig. 2).

In WD SW-fed animals, a statistically significant decrease of TCBVs after 28 weeks of high-fat diet has been observed (0.431 ± 0.01584 cm^3^, *p* < 0.05, −2.49%) (Fig. 2). Moreover, comparisons of TCBVs at specific times showed a decrease at 4 weeks (*p* < 0.01, −3.48%), 8 weeks (*p* < 0.001, −4.88%), 20 weeks (*p* < 0.001, −6.11%) and 28 weeks (*p* < 0.01, −7.44%) respectively WD SW cohort compared to the control group (Fig. 2).

### Smaller total cerebral brain volumes were associated with Steatosis and Steatohepatitis

**Figure 3:**
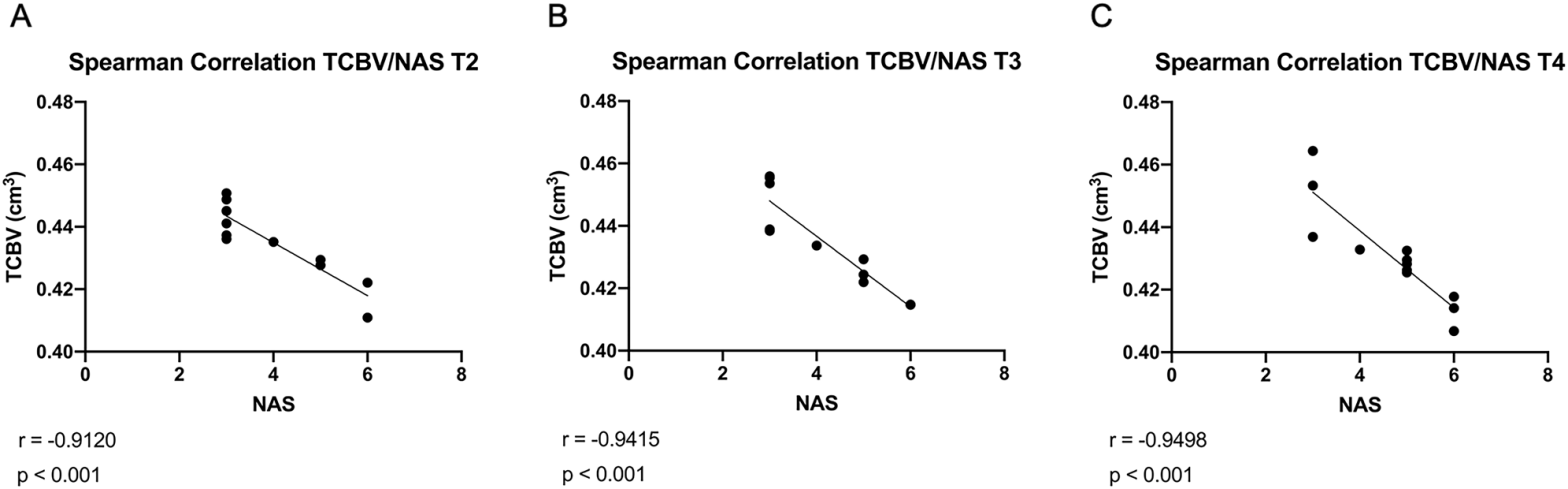
Relationship between TCBV (Total cerebral brain volume, cm^3^) and NAS (NAFLD Activity Score, 0–8) by Spearman-related analysis. Lines are generated using regression analysis (GraphPad Prism); r = Spearman’s correlation coefficient; NAS represents the sum of scores for steatosis, lobular inflammation, and ballooning; **(A)** T2: 8 weeks; **(B)** T3: 20 weeks; **(C)** T4: 28 weeks.

Smaller TCBVs were associated with high NAS (r = −0.9120; *p* < 0.001, Fig. 3A) already after 8 weeks (steatosis). An association between TCBV and MAFLD was also observed during the progression of the disease after 20 weeks (r = −0.9415; *p* < 0.001, Fig. 3B) as well as after 28 weeks (r = −0.9498; *p* < 0.001, Fig. 3C).

### Quantifying microglia and astrocytes morphology in immunofluorescent images of fixed thalamic

**Figure 4:**
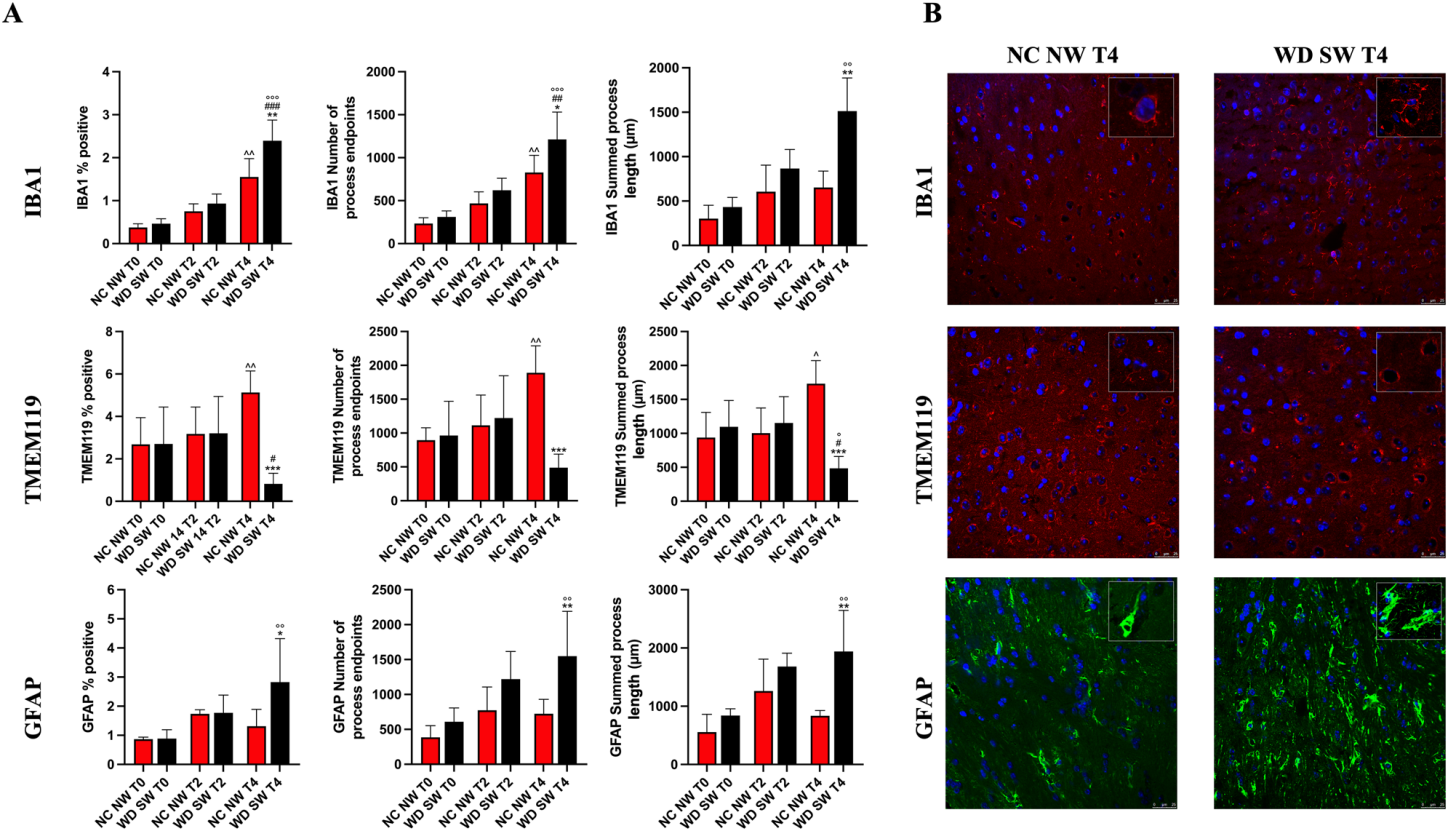
Immunofluorescence staining for IBA1 and TMEM119 in microglia and for GFAP in astrocytes. **(A)** The results are expressed as mean ± S.D. NC NW: Normal Chow Normal Water; WD SW: Western Diet Sugar Water. T0: 0 weeks; T2: 8 weeks; T4: 28 weeks. IBA1: Ionized calcium-binding adaptor molecule 1, TMEM119: Transmembrane protein 119, GFAP: Glial fibrillary acidic protein. *: *p* < 0.05, **: *p* < 0.01, ***: *p* < 0.001 vs NC NW T4; #: *p* < 0.05, ##: *p* < 0.01, ###: *p* < 0.001 vs WD SW T2, °: *p* < 0.05, °°: *p* < 0.01, °°°: *p* < 0.001 vs WD SW T0; ^: *p* < 0.05, ^^: *p* < 0.01 vs NC NW T0. **(B)** Representative confocal images of IBA1 positive microglia (above), TMEM119 positive microglia (middle), GFAP positive astrocytes (below) and magnified illustrations of microglia and astrocytes morphology.

At the baseline, immunofluorescence analysis showed the same IBA1% positive pixels, number process of endpoints and summed process length in NC NW group and WD SW animals. Likewise, no differences have been observed for TMEM119 and GFAP between NC NW and WD SW groups (Fig. 4A).

**Figure 5:**
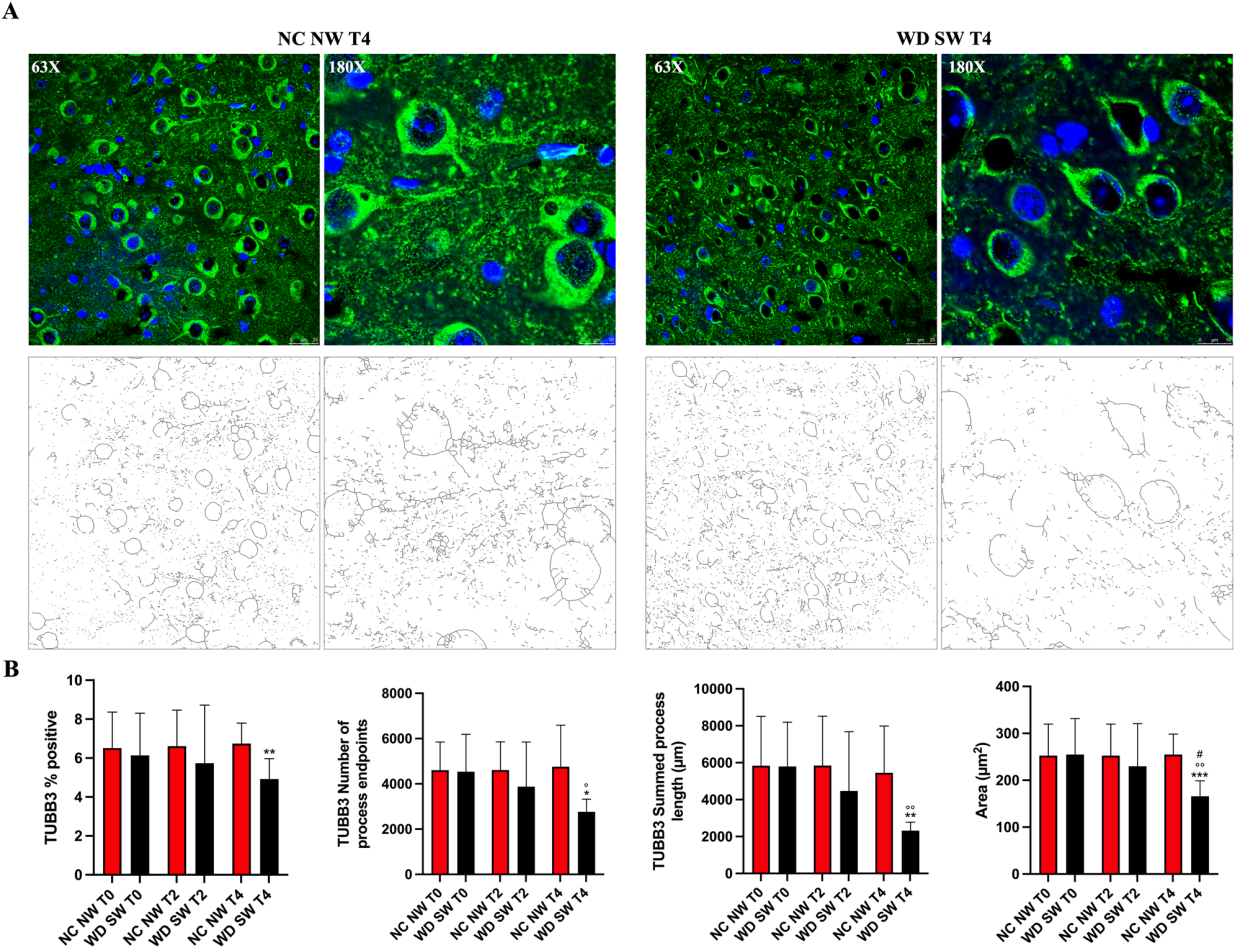
Immunofluorescence staining for TUBB3 in neurons. **(A)** Representative confocal 63X and 180X images (above) and respective 63X and 180X skeletonized images (below) of TUBB3 positive neurons at T4 (28 weeks) **(B)** The results are expressed as mean ± S.D. NC NW: Normal Chow Normal Water; WD SW: Western Diet Sugar Water. T0: 0 weeks; T2: 8 weeks; T4: 28 weeks. TUBB3: anti-β III Tubulin. *: *p* < 0.05, **: *p* < 0.01, ***: *p* < 0.001 vs NC NW T4; °: *p* < 0.05, °°: *p* < 0.01 vs WD SW T0; #: *p* < 0.05 vs WD SW T2.

IBA1% positive pixels (1.552 ± 0.4281, *p* < 0.01) and number of process endpoints (826.9 ± 200.6, *p* < 0.01) (Fig. 5A) were significant higher in NC NW group at 28 weeks compared to NC NW group at baseline (Fig. 4A).

TMEM119% positive pixels (5.127 ± 1.019, *p* < 0.01), number of process endpoints (1890 ± 398.2, *p* < 0.01), as well as summed process length (1732 ± 339.0 μm, *p* < 0.05) were significant increased after 28 weeks in NC NW group compared to NC NW group at baseline (Fig. 4A).

In the WD SW group, IBA1 showed a significant increase after 28 weeks of high-fat diet for % positive pixels (2.394 ± 0.4801, *p* < 0.001), number of process endpoints (1213 ± 318.7, *p* < 0.001) as well as summed process length (1513 ± 373.0 μm, *p* < 0.01) (Fig. 4A).

On the other hand, TMEM119 summed process length (485.2 ± 173.6 μm, *p* < 0.05) showed a statistically significant decrease after 28 weeks of high-fat diet (Fig. 4A).

Comparisons at specific times showed that IBA1% positive pixels (*p* < 0.01), number of process endpoints (*p* < 0.05) and summed process length (*p* < 0.01) were significantly higher in WD SW group than in NC NW group at 28 weeks (Fig. 4A,B), whereas TMEM119% positive (*p* < 0.001), number of process endpoints (*p* < 0.001) and summed process length (*p* < 0.001) were significantly lower in WD SW group than in NC NW group at 28 weeks (Fig. 4A,B).

In the WD SW group, the number of GFAP positive cells (2.828 ± 1.494, *p* < 0.01), number of process endpoints (1549 ± 641.3, *p* < 0.01) and summed process length (1940 ± 705.3 μm, *p* < 0.01) were significant higher after 28 weeks of high-fat diet, (Fig. 4A). Comparisons at specific times showed that GFAP % positive cells (*p* < 0.05), number of process endpoints (*p* < 0.01) and summed process length (*p* < 0.01) were significantly higher in WD SW group than in NC NW animals at 28 weeks (Fig. 4A,B).

In the WD SW group, TUBB3 showed a significant decrease after 28 weeks of high-fat diet for number of process endpoints (2761 ± 562.3, *p* < 0.05), summed process length (2323 ± 454.9, *p* < 0.01) as well as area of neurons (165.9 ± 32.76, *p* < 0.01) (Fig. 5B). Comparisons at specific times showed that TUBB3% positive cells (*p* < 0.01), number of process endpoints (*p* < 0.05), summed process length (*p* < 0.01) and area of neurons (*p* < 0.001) were significantly lower in WD SW group than in NC NW animals at 28 weeks (Fig. 5A,B).

### ^1^H-MRS of control and high-fat diet-fed animals

**Figure 6:**
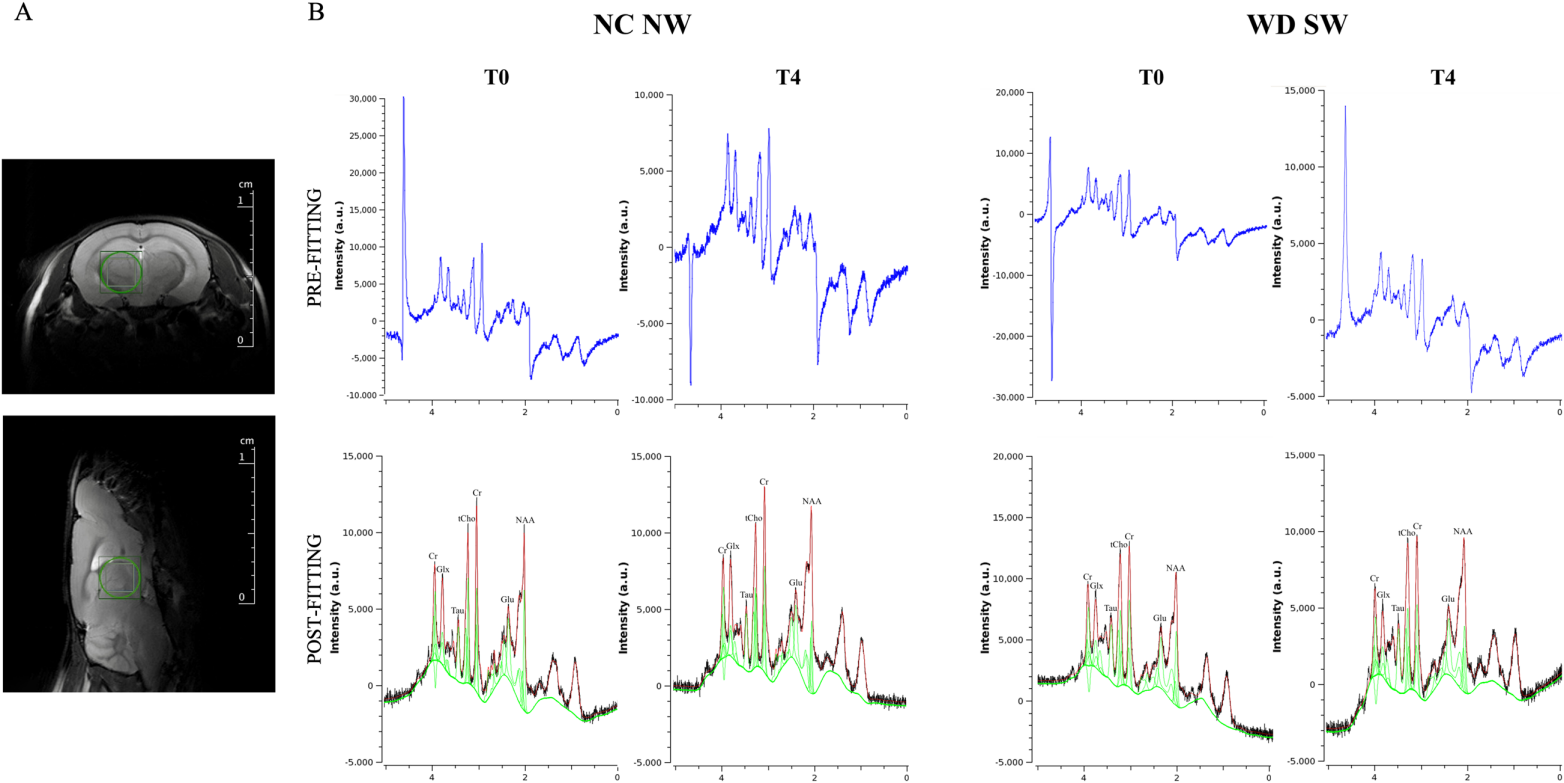
^1^H-MRS of the mouse thalamus. (**A**) Representative axial and sagittal T2_turboRARE weighted images with fat suppression of mouse brain and corresponding voxel location centered in the thalamic region (2 × 2 × 2 mm^3^). (**B**) Representative in vivo pre-fitting and post-fitting ^1^H-MRS spectra from NC NW mouse thalamus at baseline (T0) and after 28 weeks (T4) (left) and WD SW mouse thalamus at baseline (T0) and after 28 weeks (T4) (right), performed by Tarquin. In post-fitting ^1^H-MRS spectra the black trace represents the processed signal, the red trace is the model signal, while the green trace is the baseline plus the individual metabolite spectral lines.

**Figure 7:**
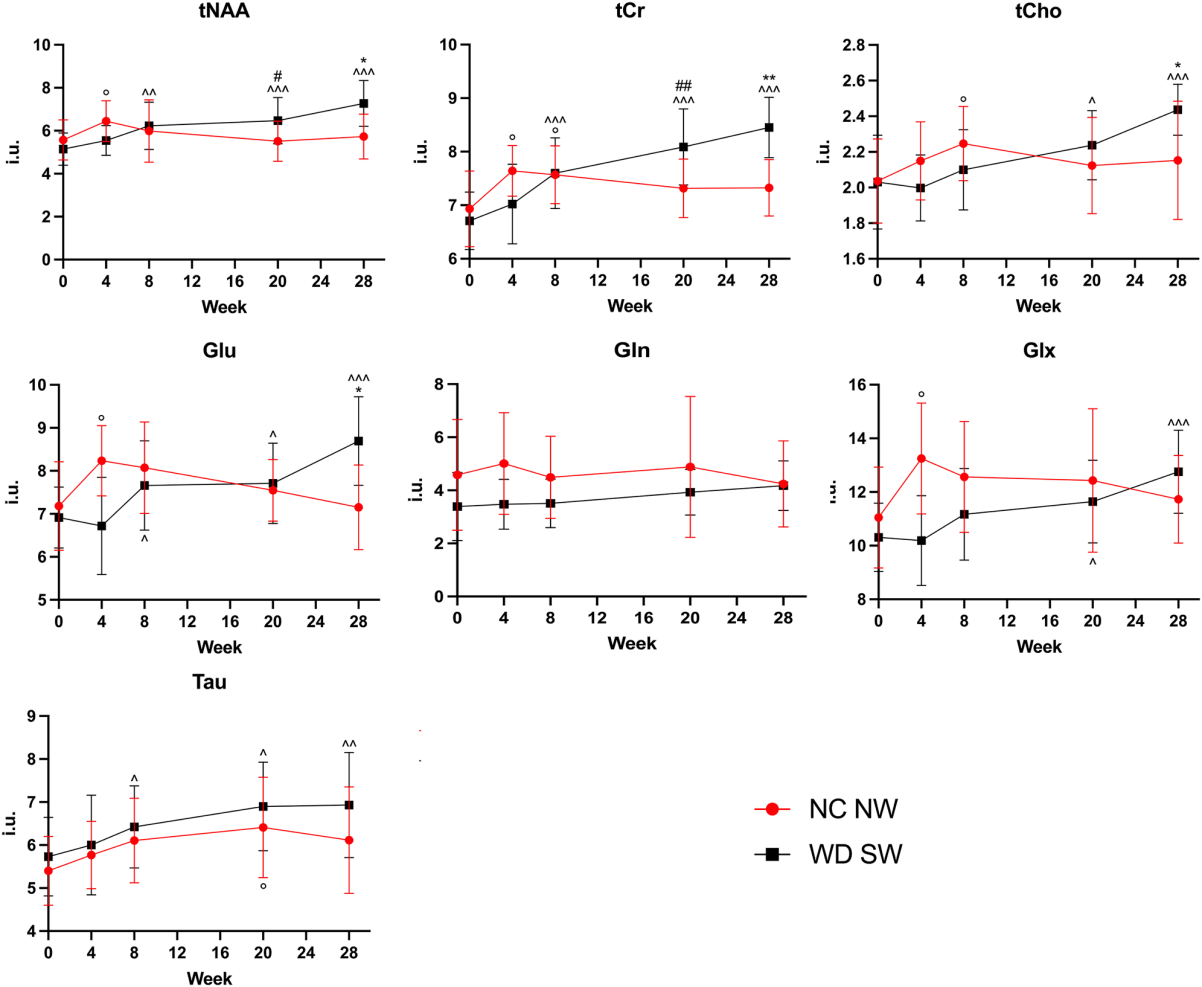
Concentration values of the metabolites for NC NW (red) and WD SW (black) mice during the 28 weeks investigated. The results are expressed as mean ± S.D. *: *p* < 0.05, **: *p* < 0.01 vs NC NW T4; #: *p* < 0.05, ##: *p* < 0.01 vs NC NW T3; ^: *p* < 0.05, ^^: *p* < 0.01, ^^^: *p* < 0.001 vs WD SW T0; °: *p* < 0.05 vs NC NW T0. i.u.: institutional units. NC NW: Normal Chow Normal Water, WD SW: Western Diet Sugar Water; T0: 0 weeks, T1: 4 weeks, T2: 8 weeks, T3: 20 weeks, T4: 28 weeks. tNAA, total N-acetylaspartate; tCr, total creatine; tCho, total choline; Glu, glutamate; Gln, Glutamine; Glx, glutamate + glutamine; Tau, Taurine.

^1^H-MRS spectra of the mouse thalamus (Fig. 6A) for the NC NW and WD SW animals underlined the presence of the main brain metabolites between 0.7 and 4 PPM (Fig. 6B and Supplementary Fig. 1A, 1B). The quantification of ^1^H-MRS spectra showed similar concentrations at the baseline in both cohorts (Fig. 7).

In the NC NW group, no significant changes occurred in metabolites concentration during the 28 weeks of the experiment, except for tNAA (6.446 ± 0.947 i.u., *p* < 0.05, + 15.67%), tCr (7.641 ± 0.4744 i.u., *p* < 0.05, + 10.21%), Glu (8.236 ± 0.8181 i.u., *p* < 0.05, + 14.70%) and Glx (13.250 ± 2.069 i.u., *p* < 0.05, + 19.91%), which were significantly higher at 4 weeks. Furthermore, the concentrations of tCr (7.569 ± 0.5391 i.u., *p* < 0.05, + 9.17%) and tCho (2.247 ± 0.2088 i.u., *p* < 0.05, + 10.31%) augmented at 8 weeks as well as the concentration of Tau (6.412 ± 1.168 i.u., *p* < 0.05, + 18.67%) at 20 weeks of diet (Fig. 7).

In the WD SW group, the concentrations of tNAA (6.23 ± 1.097 i.u., *p* < 0.01, + 21.09%), tCr (7.6 ± 0.6619 i.u., *p* < 0.001, + 13.31%), Glu (7.659 ± 1.040 i.u., *p* < 0.05, + 10.74%) and Tau (6.422 ± 0.9555 i.u., *p* < 0.05, + 12.06%) were significantly increased after 8 weeks of high-fat diet (Fig. 7). The concentrations of tNAA (6.469 ± 1.077 i.u., *p* < 0.001, + 25.73%), tCr (8.088 ± 0.709 i.u., *p* < 0.001, + 20.59%), tCho (2.238 ± 0.1946 i.u., *p* < 0.05, + 10.19%), Glu (7.711 ± 0.9338 i.u., *p* < 0.05, + 11.49%), Glx (11.64 ± 1.541 i.u., *p* < 0.05, + 12.9%) and Tau (6.897 ± 1.028 i.u., *p* < 0.05, + 29.89%) were significantly increased after 20 weeks of high-fat diet (Fig. 7).

Likewise, the concentrations of tNAA (7.269 ± 1.072 i.u., *p* < 0.001, + 41.28%), tCr (8.452 ± 0.5623 i.u., *p* < 0.001, + 26.02%), tCho (2.437 ± 0.1436 i.u., *p* < 0.001, + 19.99%), Glu (8.693 ± 1.028 i.u., *p* < 0.001, + 25.7%), Glx (12.760 ± 1.553 i.u., *p* < 0.001, + 23.76%) and Tau (6.930 ± 1.222 i.u., *p* < 0.05, + 20.92%) were significantly increased after 28 weeks of high-fat diet (Fig. 7).

Comparisons at specific times showed that the concentrations of tNAA (*p* < 0.05, + 17.32%) and tCr (*p* < 0.01, + 10.58%) were significantly higher in the WD SW group than in the NC NW control group at 20 weeks, as well as the concentrations of tNAA (*p* < 0.05, + 26.84%), tCr (*p* < 0.01, + 15.37%), tCho (*p* < 0.05, + 13.19%) and Glu (*p* < 0.05, + 21.55%) were significantly increased in the WD SW than NC NW cohort at 28 weeks (Fig. 7).

## Discussion

MAFLD includes a wide spectrum of liver diseases^1^, that can start with the accumulation of lipid molecules in hepatocytes and evolve into NASH^5,6^. Despite the main feature is liver dysfunction, the detrimental impact of lipid accumulation can affect the whole metabolic state also predisposing to cardiovascular and neurological diseases^36^. In this view, the aim of our study was to investigate the effects of the altered lipid metabolism during NASH development in the thalamus. In particular, we monitored and quantified over time the putative indicator of inflammation Taurine, and the levels of Glutamate, tNAA, tCho, tCr to study the impact of brain metabolic fluctuation on its energetic status, structure and function.

In patients suffering from MAFLD, a high-fat diet was associated with a range of systemic dysfunctions that include a gain of weight and fasting glucose, abnormal fasting insulin levels, raised lipid biosynthesis in the liver, elevated levels of circulating fatty acids and glucose intolerance^37^.

The occurrence of these events was studied in depth in an animal model of MAFLD resembling the human features of disease development^38^, confirming that chronic intake of a diet enriched in fats and carbohydrates contributes to induce inflammation and oxidative damage primarily to the hepatic microcirculation and then, at systemic level^39,40^. This suggests a more generalized endothelial dysfunction, also involving blood–brain barrier that, once damaged, permits the infiltration of circulating inflammatory cells into the brain^41^ causing neuroinflammation, oxidative stress, metabolic impairment and neurotransmitter transmission deficit^42,43^.

The occurrence of steatosis, lobular inflammation, and hepatocellular ballooning, characterizing WD SW-induced NASH in our experiments, further support the hypothesis of its possible impact on the brain, as shown by the brain volume reduction and thalamus inflammation during the progression of MAFLD. Indeed, at thalamic level, high fat diet consumption determined a progressive gliosis, revealed evaluating morphology and activity of microglia and astrocytes, respectively.

To better define the role of microglia under NASH, a microglial and macrophage-specific calcium-binding protein, named ionized calcium-binding adaptor molecule 1 (IBA1), and the transmembrane protein 119 (TMEM119), expressed on microglia-derived cells but not on recruited blood-derived macrophages^44^ were marked.

At the end of the study, our experiments show that although fat diet induced a loss of the resident microglia at thalamic level, as shown by the reduction of TMEM119 positive cells, the concomitant decrease in the process endpoint number and the summed process length highlighted an amoeboid morphology, indicative of a transformation toward the macrophage phenotype. Conversely, a significant increase of IBA1 positive cells was observed starting from 14 weeks. Before sacrifice, this raise became significant in the thalamus of MAFLD mice as compared to control; in addition, IBA1 positive cells showed an enhancement of the process endpoint number and the summed process length, suggesting the differentiation of infiltered-monocytes in a macrophagic phenotype.

Overall, these data suggest that the time-dependent activation of microglia, in mice fed a high fat diet, caused an inflammatory degeneration at thalamic level that, on the other hand, was accompanied by astrocyte activation, as shown by the enhancement of GFAP positive cells, process endpoint number and summed process length. Astrocyte reactive gliosis is recognized as highly heterogeneous state in which astrocyte activities depend on the specific injury. Indeed, they can both sustain regeneration or promote detrimental effects on surrounding cells and brain parenchyma^45^. In this view, our results need further insights because they might reflect a neuroprotective effect to counterbalance exacerbate inflammation or, differently, a contribution to the inflammatory condition amplification.

NASH-induced inflammation, in turn, probably affects brain metabolism, as shown by spectroscopic analysis. In particular, the changes in the metabolic parameters highlighted a time-dependent increase in the concentration of taurine, considered a hypothetical marker of inflammation, up to twentieth week was detected. This gradual increase was accompanied, until the end of the experiment, by an enhancement in total choline which, normally, represents the sum of the levels of glycerophosphorylcholine and phosphorylcholine, both precursors of phosphatidylcholine and sphingomyelin^31,46,47^.

Taken together, these results indicate that the solubilization of glycerophosphorylcholine and phosphorylcholine, probably due to oxidative/inflammatory insults affecting membranes, could be responsible not only for neuronal demyelination, but also for the alteration of plasma membrane permeability and polarization, and for the dysfunction of neurotransmitter vesicular release^29,48–50^.

At the end of the experiment, in thalamus of WD SW mice, increased NAA and glutamate levels were also highlighted, although they were not associated with any changes in glutamine levels. This suggests that, in the presence of increased glucose tolerance and increased levels of circulating fatty acids, as typically observed at that time point in DIAMOND mice^28^, cerebral tissue activates an alternative mechanism to the use of glucose, capable to equally satisfy its energy needs. Indeed, although glucose has always been recognized as the primary source of brain energy, growing evidence shows that other metabolites, such as glutamate and acetate, are used as energy sources, mainly by astrocytes, both in physiological and pathological conditions^51^. In this perspective, the increase in astrocytic glutamate could represent the substrate needed to an anaplerotic reaction aimed to ensure the right homeostasis of Krebs cycle and to the production of necessary lactate for neuronal survival.

The use of glutamate as a mitochondrial substrate^52–54^ in turn, could justify its vesicular depletion at synaptic level. Consequently, the lack of glutamate release, compared to the unchanged levels of glutamine measured over time, could explain the cognitive deficits characterizing NASH^55^.

In conditions of impaired metabolism, the brain can also use free fatty acids to produce energy^56^.

The main source of free fatty acids crossing the blood brain barrier may come from long-chain fatty acid/albumin complexes and, to a lesser extent, from circulating lipoproteins^56^. Once inside the astrocytes, the conversion into acetyl-CoA, operated by the acyl-CoA synthetase, allows its translocation into the mitochondrial matrix for β-oxidation and for the production of ketone bodies, such as acetoacetate, beta-hydroxybutyrate and acetone that results from their spontaneous decomposition^57^. Ketone bodies are synthesized starting from two acetyl-CoA molecules also at the peripheral level, mainly by the liver, especially in conditions of decreased glucose bioavailability. Subsequently, they are transported to the extrahepatic tissues, where they are used, after conversion into acetyl-CoA and introduction into the cycle of tricarboxylic acids, for energy production^58^.

Therefore, the ketone bodies produced by astrocytes or coming from the bloodstream in conditions of more marked metabolic alterations migrate within neurons, where they are converted into acetyl-CoA and used in the Krebs cycle. On the other hand, acetyl-CoA excess is converted into NAA and stored inside neuronal mitochondria for satisfying a possible sudden increase in energy needs^59^. This effect, aimed to preserve neuronal function, might represent a protective mechanism against fat-induced cell damage highlighted by the changes in neuronal morphology. Indeed, TUBB3 tubulin staining showed a time-dependent axon degeneration in WD SW mice, characterized by a decrease in total number of the process endpoint numbers and in the summed process length. In turn, the reduced branching, associated with a reduced soma roundness, suggests a decrease in neuronal network^60^ and a progressive shrinkage of the cell, probably preceding cell body apoptosis^61^.

In our experiments, the increased amount of NAA, found in thalamus of mice fed a high-fat diet, were also associated with a raise of creatine/phosphocreatine levels, also indicating the formation of phosphate reservoirs needed for ATP synthesis.

Overall, the production of NAA and creatine/phosphocreatine appears necessary to constantly ensure correct mitochondrial functionality and, consequently, the energy needed for brain functions potentially compromised by the inflammatory insult triggered at the peripheral level by NASH. On the other hand, the tight correlation between reduced brain volume and MAFLD development, revealed by our experiments, further supports the hypothesis of an increased risk of functional deficits of specific brain areas.

Therefore, since the thalamus represents a key element in the integration of neuronal impulses within the network including prefrontal cortex, hippocampus, amygdala and mammillary bodies, a constant energy supply must be always maintained^62^.

In this scenario, the use of newly synthesized glutamate as an energy source, rather than as a neurotransmitter reserve, could represent a key element for the compensation of the energetic deficit to prevent neuronal damage, but at the same time, the triggering cause of the learning and memory deficits that are often found in NASH affected patients^15^.

In conclusion, further studies are needed to better clarify the pathophysiological mechanisms underlying metabolic changes and inflammation development in central nervous system under NASH. The explanation of these aspects might also highlight common features with other neuroinflammatory disease, such as multiple sclerosis and Alzheimer disease.

## Supplementary Information

Supplementary Information.

## References

[1] Eslam, M., Sanyal, A. J., George, J., International consensus panel. MAFLD: a consensus-driven proposed nomenclature for metabolic associated fatty liver disease. *Gastroenterology 158*, 1999–2014.e1 (2020). 10.1053/j.gastro.2019.11.312 32044314

[2] Fouad Y, Elwakil R, Elsahhar M (2021). The NAFLD-MAFLD debate: eminence vs evidence. Liver Int..

[3] Chen Y, Li H, Li S (2021). Prevalence of and risk factors for metabolic associated fatty liver disease in an urban population in China: a cross-sectional comparative study. BMC Gastroenterol..

[4] Estes C, Razavi H, Loomba R, Younossi Z, Sanyal AJ (2018). Modeling the epidemic of nonalcoholic fatty liver disease demonstrates an exponential increase in burden of disease. Hepatology.

[5] Kleiner DE (2005). Design and validation of a histological scoring system for nonalcoholic fatty liver disease. Hepatology.

[6] Musolino V, Gliozzi M, Scarano F (2020). Bergamot polyphenols improve dyslipidemia and pathophysiological features in a mouse model of non-alcoholic fatty liver disease. Sci. Rep..

[7] Guzman G (2008). Does nonalcoholic fatty liver disease predispose patients to hepatocellular carcinoma in the absence of cirrhosis. Arch. Pathol. Lab. Med..

[8] Bélanger M, Allaman I, Magistretti PJ (2011). Brain energy metabolism: focus on astrocyte-neuron metabolic cooperation. Cell Metab..

[9] Lombardi R, Fargion S, Fracanzani AL (2019). Brain involvement in non-alcoholic fatty liver disease (NAFLD): a systematic review. Dig. Liver Dis..

[10] Abdeldyem, S. M., Goda, T., Khodeir, S. A., Abou Saif, S. & Abd-Elsalam, S. Nonalcoholic fatty liver disease in patients with acute ischemic stroke is associated with more severe stroke and worse outcome. *J. Clin. Lipidol. 11*, 915–919 (2017). 10.1016/j.jacl.2017.04.115 28579247

[11] Mahfood Haddad, T., Hamdeh, S., Kanmanthareddy, A. & Alla, V. M. Nonalcoholic fatty liver disease and the risk of clinical cardiovascular events: a systematic review and meta-analysis. *Diabetes Metab. Syndr. 11 Suppl 1*, S209–S216 (2017). 10.1016/j.dsx.2016.12.033 28017631

[12] García-Ruiz C, Fernández-Checa JC (2018). Mitochondrial oxidative stress and antioxidants balance in fatty liver disease. Hepatol. Commun..

[13] Paradies G, Paradies V, Ruggiero FM, Petrosillo G (2014). Oxidative stress, cardiolipin and mitochondrial dysfunction in nonalcoholic fatty liver disease. World J. Gastroenterol..

[14] Desjardins P, Butterworth RF (2005). Role of mitochondrial dysfunction and oxidative stress in the pathogenesis of selective neuronal loss in Wernicke’s encephalopathy. Mol. Neurobiol..

[15] Higarza, S. G. *et al.* Neurobehavioral dysfunction in non-alcoholic steatohepatitis is associated with hyperammonemia, gut dysbiosis, and metabolic and functional brain regional deficits. *PLoS ONE 14*, e0223019 (2019). 10.1371/journal.pone.0223019 PMC6754158 31539420

[16] Daniele, G. *et al.* Plasma N-acetylaspartate is related to age, obesity, and glucose metabolism: effects of antidiabetic treatment and bariatric surgery. *Front. Endocrinol. (Lausanne) 11*, 216 (2020). 10.3389/fendo.2020.00216 PMC7181885 32362872

[17] Beal MF (2011). Neuroprotective effects of creatine. Amino Acids.

[18] Zheng Y, Yang Y, Dong B (2016). Metabonomic profiles delineate potential role of glutamate-glutamine cycle in *db/db*mice with diabetes-associated cognitive decline. Mol. Brain.

[19] Shivaraj, M. C. *et al.* Taurine induces proliferation of neural stem cells and synapse development in the developing mouse brain. *PLoS ONE 7*, e42935 (2012). 10.1371/journal.pone.0042935 PMC3423436 22916184

[20] Sonnay S, Gruetter R, Duarte JMN (2017). How energy metabolism supports cerebral function: insights from 13C magnetic resonance studies in vivo. Front. Neurosci..

[21] Lizarbe B, Soares AF, Larsson S, Duarte JMN (2018). Neurochemical modifications in the hippocampus, cortex and hypothalamus of mice exposed to long-term high-fat diet. Front. Neurosci..

[22] Horvath TL (2010). Synaptic input organization of the melanocortin system predicts diet-induced hypothalamic reactive gliosis and obesity. Proc. Natl. Acad. Sci. USA.

[23] Weinstein G (2018). Association of nonalcoholic fatty liver disease with lower brain volume in healthy middle-aged adults in the framingham study. JAMA Neurol..

[24] Williamson JB (2020). Cerebral metabolite concentrations are associated with cortical and subcortical volumes and cognition in older adults. Front. Aging Neurosci..

[25] Zahr N, Mayer D, Vinco S (2009). *In Vivo* evidence for alcohol-induced neurochemical changes in rat brain without protracted withdrawal, pronounced thiamine deficiency, or severe liver damage. Neuropsychopharmacology.

[26] Hoon AH, Belsito KM, Nagae-Poetscher LM (2003). Neuroimaging in spasticity and movement disorders. J. Child Neurol..

[27] Xu S (2013). In vivo high-resolution localized (1) H MR spectroscopy in the awake rat brain at 7 T. Magn. Reson. Med..

[28] Asgharpour A (2016). A diet-induced animal model of non-alcoholic fatty liver disease and hepatocellular cancer. J. Hepatol..

[29] Aureli M, Grassi S, Prioni S, Sonnino S, Prinetti A (2015). Lipid membrane domains in the brain. Biochim. Biophys. Acta.

[30] Jakaria M (2019). Taurine and its analogs in neurological disorders: Focus on therapeutic potential and molecular mechanisms. Redox. Biol..

[31] Lizarbe B, Cherix A, Duarte JMN (2019). High-fat diet consumption alters energy metabolism in the mouse hypothalamus. Int. J. Obes..

[32] Albrecht J, Sidoryk-Węgrzynowicz M, Zielińska M, Aschner M (2010). Roles of glutamine in neurotransmission. Neuron Glia Biol..

[33] Wilson M, Reynolds G, Kauppinen RA, Arvanitis TN, Peet AC (2011). A constrained least-squares approach to the automated quantitation of in vivo ^1^H magnetic resonance spectroscopy data. Magn. Reson. Med..

[34] Siddiqui MS (2018). Case definitions for inclusion and analysis of endpoints in clinical trials for nonalcoholic steatohepatitis through the lens of regulatory science. Hepatology.

[35] Bedossa P (2012). Histopathological algorithm and scoring system for evaluation of liver lesions in morbidly obese patients. Hepatology.

[36] Musolino V (2020). The synergistic effect of *Citrus bergamia* and *Cynara cardunculus* extracts on vascular inflammation and oxidative stress in non-alcoholic fatty liver disease. J. Tradit. Compl. Med..

[37] Younossi ZM (2019). The global epidemiology of NAFLD and NASH in patients with type 2 diabetes: a systematic review and meta-analysis. J. Hepatol..

[38] Castro RE, Diehl AM (2018). Towards a definite mouse model of NAFLD. J. Hepatol..

[39] Gliozzi M (2021). Cholesterol homeostasis: researching a dialogue between the brain and peripheral tissues. Pharmacol. Res..

[40] Scarano F (2021). Potential of nutraceutical supplementation in the modulation of white and brown fat tissues in obesity-associated disorders: role of inflammatory signalling. Int. J. Mol. Sci..

[41] Mondal A (2020). Lipocalin 2 induces neuroinflammation and blood-brain barrier dysfunction through liver-brain axis in murine model of nonalcoholic steatohepatitis. J. Neuroinflamm..

[42] Lyn-Cook LE (2009). Hepatic ceramide may mediate brain insulin resistance and neurodegeneration in type 2 diabetes and non-alcoholic steatohepatitis. J. Alzheimers Dis..

[43] de la Monte SM, Longato L, Tong M, Wands JR (2009). Insulin resistance and neurodegeneration: roles of obesity, type 2 diabetes mellitus and non-alcoholic steatohepatitis. Curr. Opin. Investig. Drugs.

[44] Zrzavy T (2017). Loss of 'homeostatic' microglia and patterns of their activation in active multiple sclerosis. Brain.

[45] Hyvärinen T (2019). Co-stimulation with IL-1β and TNF-α induces an inflammatory reactive astrocyte phenotype with neurosupportive characteristics in a human pluripotent stem cell model system. Sci. Rep..

[46] Oshida K (2003). Effects of dietary sphingomyelin on central nervous system myelination in developing rats. Pediatr. Res..

[47] Li YH, Woo SH, Choi DH, Cho EH (2015). Succinate causes α-SMA production through GPR91 activation in hepatic stellate cells. Biochem. Biophys. Res. Commun..

[48] Krols M, Bultynck G, Janssens S (2016). ER-mitochondria contact sites: a new regulator of cellular calcium flux comes into play. J. Cell Biol..

[49] Lauwers E, Goodchild R, Verstreken P (2016). Membrane lipids in presynaptic function and disease. Neuron.

[50] van Echten-Deckert G, Alam S (2018). Sphingolipid metabolism: an ambiguous regulator of autophagy in the brain. Biol. Chem..

[51] Moffett JR, Arun P, Ariyannur PS, Namboodiri AM (2013). N-Acetylaspartate reductions in brain injury: impact on post-injury neuroenergetics, lipid synthesis, and protein acetylation. Front. Neuroenerget..

[52] Bowser TE, Trawick ML (2013). Probing the specificity of gamma-glutamylamine cyclotransferase: an enzyme involved in the metabolism of transglutaminase-catalyzed protein crosslinks. Amino Acids.

[53] Norenberg MD (1979). Distribution of glutamine synthetase in the rat central nervous system. J. Histochem. Cytochem..

[54] Bernstein HG (2015). Reduced density of glutamine synthetase immunoreactive astrocytes in different cortical areas in major depression but not in bipolar I disorder. Front. Cell Neurosci..

[55] Limón ID, Angulo-Cruz I, Sánchez-Abdon L, Patricio-Martínez A (2021). Disturbance of the glutamate-glutamine cycle, secondary to hepatic damage, compromises memory function. Front. Neurosci..

[56] Schönfeld P, Reiser G (2013). Why does brain metabolism not favor burning of fatty acids to provide energy? Reflections on disadvantages of the use of free fatty acids as fuel for brain. J. Cereb. Blood Flow. Metab..

[57] McPherson PA, McEneny J (2012). The biochemistry of ketogenesis and its role in weight management, neurological disease and oxidative stress. J. Physiol. Biochem..

[58] Puchalska P, Crawford PA (2017). Multi-dimensional roles of ketone bodies in fuel metabolism, signaling, and therapeutics. Cell Metab..

[59] Romano A (2017). Fats for thoughts: an update on brain fatty acid metabolism. Int. J. Biochem. Cell Biol..

[60] Pani G (2013). Morphological and physiological changes in mature in vitro neuronal networks towards exposure to short-, middle- or long-term simulated microgravity. PLoS ONE.

[61] Chi H, Chang HY, Sang TK (2018). Neuronal cell death mechanisms in major neurodegenerative diseases. Int. J. Mol. Sci..

[62] Panov A, Orynbayeva Z, Vavilin V, Lyakhovich V (2014). Fatty acids in energy metabolism of the central nervous system. Biomed. Res. Int..

