# Supplementary material for: MAFLD progression contributes to altered thalamus metabolism and brain structure
Source: Sci Rep. 2022 Jan 24;12:1207. doi: 10.1038/s41598-022-05228-5 (PMC8786899; doi:10.1038/s41598-022-05228-5)
Supplement: Supplementary file 1 — Supplementary Information. [file 41598_2022_5228_MOESM1_ESM.docx]

**MAFLD PROGRESSION CONTRIBUTES TO ALTERED THALAMUS METABOLISM AND BRAIN STRUCTURE**

Saverio Nucera^1*^, Stefano Ruga^1*^, Antonio Cardamone^1#^, Anna Rita Coppoletta^1#^, Lorenza Guarnieri^1^, Maria Caterina Zito^1^, Francesca Bosco^1^, Roberta Macrì^1^, Federica Scarano^1^, Miriam Scicchitano^1^, Jessica Maiuolo^1^, Cristina Carresi^1^, Rocco Mollace^1^, Luca Cariati^1^, Giuseppe Mazzarella^2^, Ernesto Palma^1^, Micaela Gliozzi^1^^†^, Vincenzo Musolino^1^, Giuseppe Lucio Cascini^2^, Vincenzo Mollace^1^

1. Institute of Research for Food Safety & Health IRC-FSH, University Magna Graecia, 88100 Catanzaro, Italy.

2. Nuclear Medicine Unit, Department of Diagnostic Imaging, Magna Graecia University, 88100 Catanzaro, Italy.

*Equally contribution

#Equally contribution

†Correspondence: gliozzi@unicz.it (M.G.)

**SUPPLEMENTARY RESULTS**

**^1^**H-MRS spectra of the mouse thalamus belonging to the NC NW and WD SW groups underlined the presence of the main brain metabolites between 0.7 – 4 PPM (Fig. **6B** **and Supplementary Figure 1A, 1B**). *In vivo* pre-fitting and post-fitting ^1^H-MRS spectra from a NC NW mouse and a mouse fed a high fat diet are shown at all time points (T0/T1/T2/T3/T4).

**B**

**A**


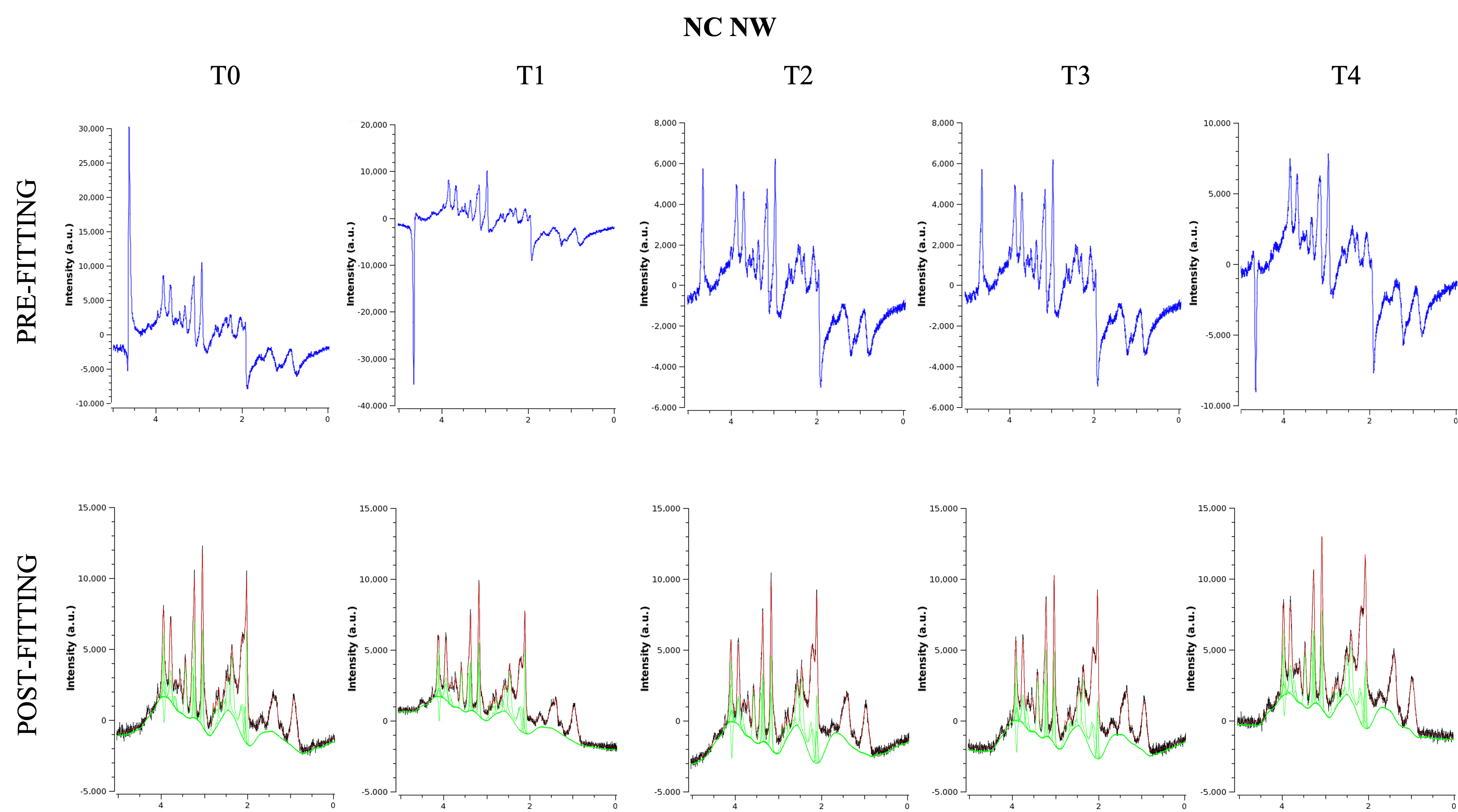

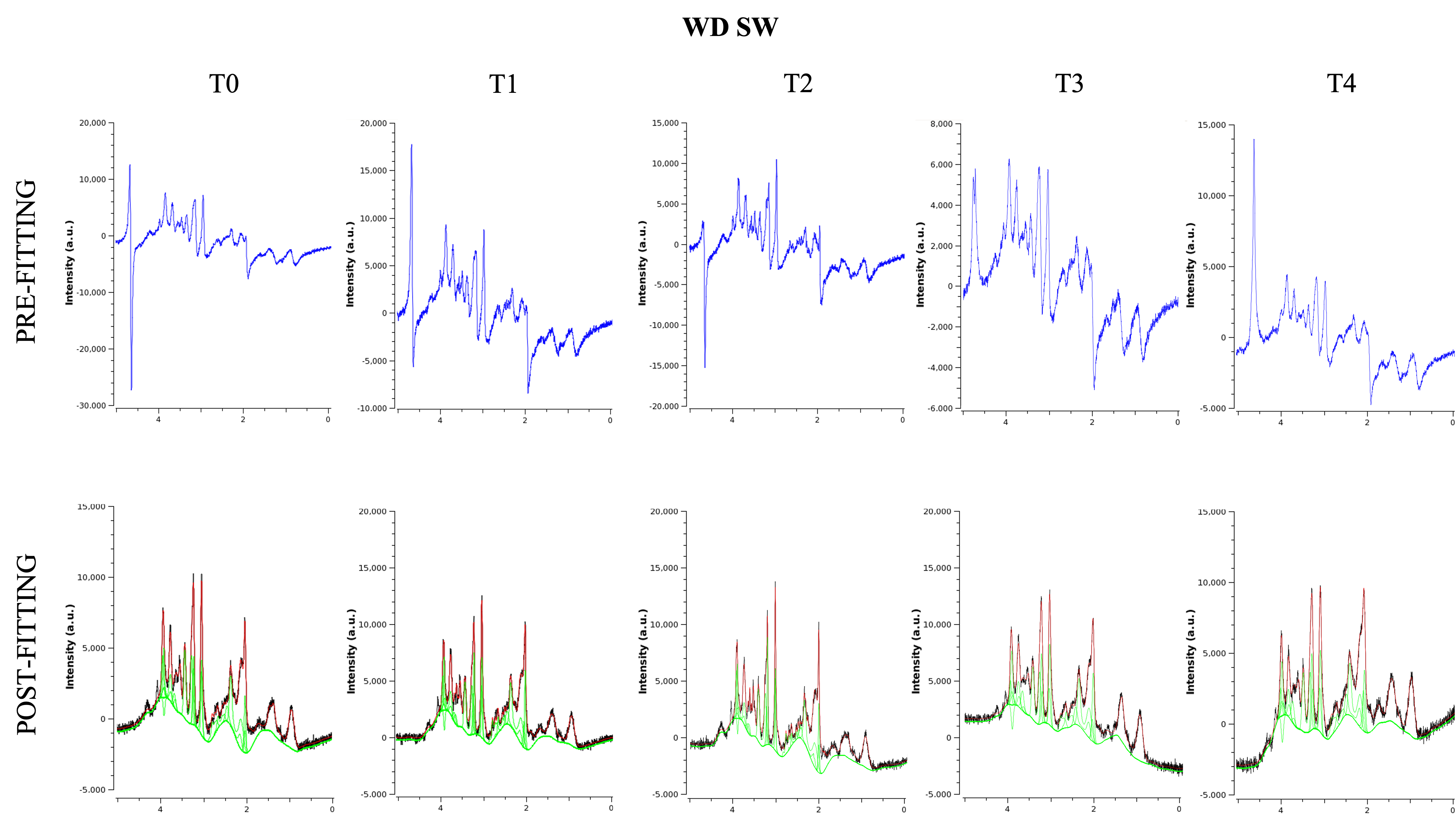


**Figure S1.** Representative in vivo pre-fitting and post-fitting ^1^H-MRS spectra from NC NW mouse and WD SW mouse at all time points (T0/T1/T2/T3/T4) performed by Tarquin.
